# BiOBr/Coal Gangue-Based SAPO-5 Molecular Sieve Nanocomposite for Enhanced Adsorption and Photocatalytic Degradation of Methylene Blue

**DOI:** 10.3390/nano15050321

**Published:** 2025-02-20

**Authors:** Boyang Xu, Jie Chen, Kai Wang, Pengfei Li, Le Kang, Huiling Du, Qianqian Liu, Xiaoqing Lian

**Affiliations:** 1 College of Materials Science and Engineering, Xi’an University of Science and Technology, Xi’an 710054, China; xuboyanglwz@163.com (B.X.); lipengfei34543@163.com (P.L.); hldu@xust.edu.cn (H.D.); liuqq@xust.edu.cn (Q.L.); lianxiaoqing@126.com (X.L.); 2 School of Electrical Engineering, Qingdao University, Qingdao 266071, China; wkwj888@163.com

**Keywords:** dye removal, coal gangue, SAPO-5 molecular sieve, photocatalyst, BiOBr

## Abstract

The accumulation of organic pollutants and solid waste is one of the major environmental challenges faced globally. Establishing an efficient recycling system for solid waste and designing cost-effective, high-performance photocatalysts are urgent tasks for the removal of organic pollutants from water. This study utilizes coal gangue as the precursor to synthesize a coal gangue-based phosphorus–silicon–aluminum molecular sieve (SAPO-5) via hydrothermal synthesis. The resulting material was then composited with bismuth oxybromide (BiOBr) to form a novel BiOBr/coal gangue-based SAPO-5 nanocomposite. When the mass ratio of BiOBr to coal gangue-based SAPO-5 molecular sieve is 0.3, the synthesized nanocomposite exhibits excellent adsorption and photocatalytic performance for the removal of methylene blue, achieving a removal rate of 97.8% and the mineralization rate of 57.4% within 30 min. The superior performance can be attributed to the optimal pore size, rapid charge transfer rate, and high photogenerated charge density of the BiOBr/coal gangue-based SAPO-5 nanocomposite. The novel BiOBr/coal gangue-based SAPO-5 molecular sieve nanocomposite catalyst presents a new approach for the harmless treatment of organic dye wastewater and the high-value utilization of coal gangue.

## 1. Introduction

With the development of the textile dyeing industry, dyes have become essential components in the dyeing process. Dyes are characterized by chemical stability, complex structures, and difficulty in degradation. Current methods for treating dye wastewater primarily include coagulation–decolorization, adsorption–extraction, ion exchange decolorization, physico-chemical-biological hybrid processes, and oxidation methods [1,2]. Compared to conventional methods for removing waterborne pollutants, photocatalytic technology can generate highly oxidative species such as holes (h^+^), hydroxyl radicals (·OH), and superoxide radicals (·O_2_^−^), which are capable of degrading most pollutants. This process avoids secondary pollution, operates under ambient temperature and pressure, requires simple equipment, and is cost-effective, making it widely applied [3,4].

Bismuth oxybromide (BiOBr) is a layered semiconductor with a moderate band gap and is widely used due to its availability and low cost. BiOBr exhibits a typical layered structure, consisting of alternating [Bi_2_O_2_]^2+^ layers and bromide ions [5]. This layered structure imparts an internal electric field, which facilitates the separation of photogenerated charge carriers. Under illumination, electrons in the valence band are excited to the conduction band, generating photogenerated electron-hole pairs. These can directly oxidize pollutant molecules, while the photogenerated electrons react with surrounding oxygen molecules to produce reactive oxygen species. These active species work synergistically to gradually decompose organic pollutants into harmless small molecules, ultimately achieving pollutant degradation [6]. Yu [7] prepared ZnO/biochar nanocomposites for the degradation of methylene blue (MB). The ZnO/biochar nanocomposites achieved a degradation rate of 95.19% for MB within 225 min. Yu [8] synthesized ZnO/N, O-type biochar nanocomposites, which achieved a degradation rate of 96% for MB within 70 min and 97.8% for tetracycline hydrochloride (TC) within 140 min.

Coal gangue (CG), as the main byproduct of coal mining, is generated in large quantities with low utilization, resulting in a significant environmental burden. Its comprehensive utilization has become a key research focus in the fields of environmental and energy studies [9]. CG is rich in silicates, aluminates, and small amounts of iron oxides, with the highest concentrations of silicon and aluminum, making it suitable for the preparation of molecular sieves. Molecular sieves are porous materials with a three-dimensional framework, and their porous structure imparts selective adsorption and separation properties [10].

Phosphorus–silicon–aluminum (SAPO) molecular sieves are commonly used as catalysts in reactions such as isomerization and alkylation [11]. Studies have shown that under UV irradiation, the active sites in SAPO-5 molecular sieves are activated, exhibiting charge transfer excitation of [Al^2+^-O^−^] *. In these active sites, Al acts as an electron acceptor, while O serves as an electron donor, imparting photocatalytic activity [12]. Qiu [13] synthesized SAPO-5/g-C_3_N_4_ heterojunction composites for photocatalytic degradation of Rhodamine B (RhB) under visible light. The degradation efficiency of the SAPO-5/g-C_3_N_4_ composite was 40.6% higher than that of pure g-C_3_N_4_.

This study uses CG as a source of silica and alumina, aluminum isopropoxide as an additional aluminum source, and phosphoric acid as the phosphorus source. Through a hydrothermal method, a fully solid waste coal gangue-based SAPO-5 molecular sieve was greenly synthesized. This was then composited with BiOBr to produce a BiOBr/coal gangue-based SAPO-5 nanocomposite with excellent adsorption and photocatalytic properties. A novel approach for efficiently separating photogenerated electron-hole pairs and degrading organic dye wastewater is proposed. The study also explores the coupling mechanism between the pore structure of BiOBr/coal gangue-based SAPO-5 nanocomposites and their photocatalytic activity for treating organic dye wastewater. This study provides a new theoretical and experimental foundation for the high-value utilization of CG waste and the treatment of dye wastewater, offering significant implications for the sustainable development of the ecological environment.

## 2. Materials and Methods

### 2.1. Materials

The CG used as the raw material was supplied by the Shenhua Shendong Coal Group, located in Shaanxi, China. Aluminum isopropoxide (C_9_H_21_AlO_3_, AR, ≥99.0%) was purchased from Yangzhou Suoao New Materials Co., Ltd., located in Yangzhou, China. Phosphoric acid (H_3_PO_4_, AR, ≥85.0%) was purchased from Hebei Pengfa Chemical Co., Ltd., located in Cangzhou, China. Triethylamine (TEA) (C_6_H_15_N, AR, ≥99.9%) was provided by Shandong Keyuan Biochemical Co., Ltd., located in Hezhe, China. Hydrofluoric acid (HF, AR, ≥40.0%) was purchased from Xilong Scientific Co., Ltd., located in Shantou, China. Bismuth nitrate pentahydrate (Bi(NO_3_)_3_⋅5H_2_O, AR, >99.0%), sodium bromide (NaBr, ACS, ≥99%), and mannitol (C_6_H_14_O_6_, AR, 98%) were purchased from Shanghai Aladdin Biochemical Technology Co., Ltd., located in Shanghai, China. Tert-butyl alcohol (t-BuOH), quinone (BQ), and triethanolamine (TEOA) were used as received, without further purification. Deionized water was prepared in-house for the experiments.

### 2.2. Characterization

The chemical composition of CG was quantitatively analyzed using X-ray fluorescence spectroscopy (XRF, PANalytical AxiosmX, Almelo, Netherlands). The crystal structure of the material was determined using a X-ray diffractometer (XRD, Bruker Corporation, Bruker D8 Advance, Marburg, Germany), with a scanning speed of 5°/min and a scanning range of 5~55°. The microstructure and surface morphology of the samples were examined using a field emission scanning electron microscope (SEM, TESCAN MIRA4, Brno, Czech Republic), and the elemental composition was determined using an energy dispersive spectrometer (EDS, Xplore 30, Amsterdam, Netherlands). The specific surface area and pore size distribution of the samples were obtained using a Micromeritics ASAP 2020 analyzer (Micromeritics, ASAP 2020, Norcross, Georgia, GA, USA) based on N_2_ adsorption–desorption isotherms at 77 K. The chemical states of the surface atoms of the material were determined using an X-ray photoelectron spectrometer (XPS, Thermo Fisher Escalab 250Xi, Waltham, Massachusetts, MA, USA). The structure and composition of the material were analyzed using a Fourier transform infrared spectrometer (FT-IR, Thermo Fisher Nicolet SummitX NicoletiS10, Waltham, Massachusetts, MA, USA). Ultraviolet-visible diffuse reflectance spectra (DRS) were collected in the range of 200-800 nm using a UV-3600i Plus spectrophotometer (Shimadzu Corporation, Kyoto, Japan) to measure the diffuse reflectance spectra. The recombination rate of electron-hole pairs in the material was characterized using a fluorescence spectrophotometer (PL, RF6000, Shimadzu Corporation, Kyoto, Japan). The molecular structure of the material was characterized using a Raman spectrometer (Raman, Xplora Plus, Horiba Scientific, Kyoto, Japan). The reactive species generated under light irradiation were characterized using an electron paramagnetic resonance spectrometer (EPR, Bruker EMX-nano, Bruker Corporation, Billerica, Massachusetts, MA, USA). Testing the changes of organic carbon content in MB solution before and after photocatalysis in total organic carbon analyzer (TOC, TOC-lcph, Shimadzu Corporation, Kyoto, Japan).

### 2.3. Preparation of BiOBr/Coal Gangue-Based SAPO-5 Nanocomposite

Prior to the synthesis of a coal gangue-based SAPO-5 molecular sieve, the CG was subjected to calcination pretreatment. The preparation of the coal gangue-based SAPO-5 molecular sieve is as follows: the CG was ground and sieved, then calcined at 800 °C for 6 h in a muffle furnace to obtain the pretreated coal gangue (PCG). Aluminum isopropoxide was pre-hydrolyzed for 12 h. Then, following the molar ratio of 0.3 SiO_2_:Al_2_O_3_:P_2_O_5_:1.3 TEA:0.2 HF:40 H_2_O, H_3_PO_4_, PCG, TEA, and HF were sequentially added (the chemical composition of CG and PCG is shown in Table S1). After aging for a period, the mixture was placed in a stainless steel autoclave lined with PTFE and subjected to hydrothermal synthesis at 200 °C for 24 h. After natural cooling, the product was washed to a pH of 7, dried, and then calcined in a muffle furnace at 550 °C for 5 h in air, resulting in a coal gangue-based SAPO-5 molecular sieve, denoted as A-SAPO-5. The final calcination was then carried out under a nitrogen atmosphere to obtain a coal gangue-based SAPO-5 molecular sieve enriched with oxygen vacancies, denoted as N-SAPO-5.

1 mmol of Bi(NO_3_)_3_⋅5H_2_O was dissolved in 25 mL of 0.1 M mannitol solution, and 1 mmol of NaBr was dissolved in 10 mL of deionized water. The two solutions were mixed thoroughly and stirred for 30 min. The mixture was then transferred to a stainless steel autoclave lined with PTFE and reacted at 180 °C for 3 h. After cooling to room temperature, the product was washed with ethanol and deionized water, then dried under vacuum at 60 °C for 12 h. Finally, the BiOBr was obtained by grinding and sieving.

**Figure 1:**
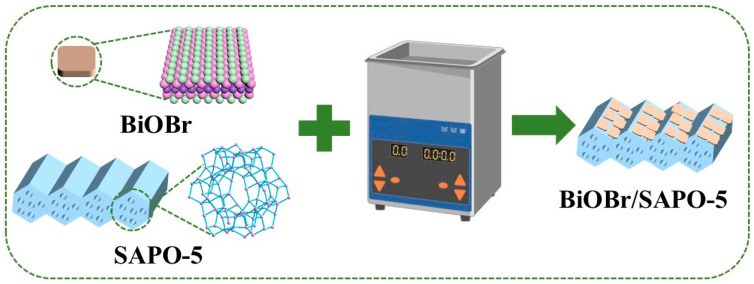
Synthesis flowchart of BiOBr/coal gangue-based SAPO-5 nanocomposite.

The prepared SAPO-5 molecular sieve was composited with BiOBr via ultrasonic impregnation to obtain the BiOBr/coal gangue-based SAPO-5 nanocomposite. A series of BiOBr/coal gangue-based SAPO-5 nanocomposites were synthesized by varying the mass ratio of BiOBr to A-SAPO-5 or N-SAPO-5 (0.2, 0.3, 0.4). The resulting materials were labeled as BA-0.2, BA-0.3, BA-0.4, BN-0.2, BN-0.3, and BN-0.4. The synthesis process of the BiOBr/SAPO-5 nanocomposite is illustrated in Figure 1.

### 2.4. Adsorption and Photocatalytic Properties

#### 2.4.1. Adsorption Experiment

The adsorption experiment was conducted at 25 ± 1 °C to evaluate the adsorption performance of the samples using methylene blue (MB) as the target pollutant. Prior to the adsorption-photocatalysis tests, the optimal sample dosage (0.2 g/L), initial concentration (100 mg/L), and pH (pH = 6) were determined (Figure S1). The experiment was conducted as follows: 20 mg of the sample was added to 100 mL of MB solution with an initial concentration of 100 mg/L, and the mixture was stirred for adsorption. At 20-min intervals, samples were withdrawn and centrifuged, and the supernatant was analyzed using a UV-Vis spectrophotometer at 665 nm to measure the concentration. The adsorption capacity was calculated using the appropriate formula, and the adsorption process was analyzed by fitting adsorption kinetics and isotherms (Supporting Information, Equations (S1)–(S7)).

#### 2.4.2. Photocatalytic Experiment

The photocatalytic performance of BiOBr/coal gangue-based SAPO-5 nanocomposites was evaluated using a photocatalytic reaction system. Test the photocatalytic activity of the sample in a photochemical reactor (Xe lamp, 300 W, radiant intensity: 230 W/m^2^, no cut-off filters, no IR-filter). The optimal sample concentration (0.2 g/L), initial concentration (100 mg/L), and pH (pH = 6) were determined (Figure S1). The experimental procedure was as follows: 20 mg of the sample was added to 100 mL of a 100 mg/L MB solution. The system was maintained in the dark for 100 min to achieve adsorption–desorption equilibrium. Afterward, the Xe lamp was turned on for illumination. At 5-minute intervals, samples were taken and centrifuged, and the supernatant was analyzed for concentration using a UV-Vis spectrophotometer at 665 nm. The degradation efficiency (*D_t_*) was calculated according to Equation (1).

*D_t_* = [(*C*_0_ − *C_t_*)/*C*_0_] × 100% (1)

The degradation behavior of the pollutant was simulated using a first-order kinetic model, as given by Equation (2).

*ln*(*C*_0_/*C_t_*) = *k*_1_*t* (2)

In the equation, *C*_0_ and *C_t_* represent the pollutant concentrations at time *t* = 0 and at time *t*, respectively.

The assessment of the cycling stability of BiOBr/coal gangue-based SAPO-5 nanocomposites is equally important. After the catalytic experiment, the photocatalyst was recovered by centrifugation, washed five times with deionized water, and dried before being subjected to further photocatalytic tests. To determine the active species involved in the photocatalytic process, scavengers such as t-BuOH, BQ, and TEOA were used to capture ·OH, ·O_2_^−^, and h^+^, respectively.

## 3. Results and Discussion

### 3.1. Structural Characterization of BiOBr/Coal Gangue-Based SAPO-5 Nanocomposites

**Figure 2:**
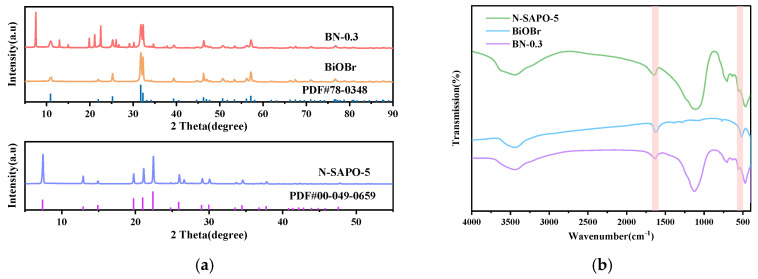

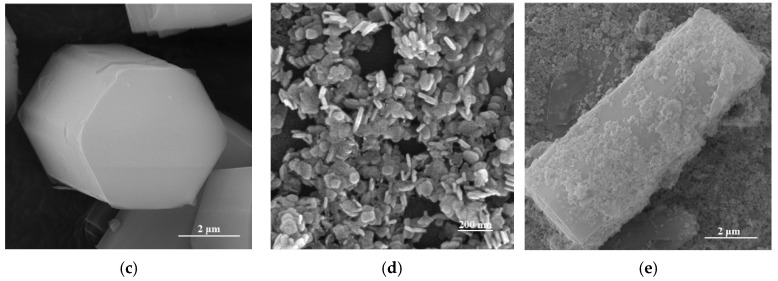
(**a**) XRD patterns of N-SAPO-5 molecular sieve, BiOBr, and BN-0.3 nanocomposite; (**b**) FT-IR spectra of N-SAPO-5 molecular sieve, BiOBr, and BN-0.3 nanocomposite; (**c**) SEM image of N-SAPO-5 molecular sieve; (**d**) SEM image of BiOBr; (**e**) SEM image of BN-0.3 nanocomposite.

Figure 2a presents the XRD patterns of the N-SAPO-5 molecular sieve, BiOBr, and BN-0.3. Comparing the diffraction peaks with the standard PDF cards, PDF#00-049-0659 (SAPO-5) and PDF#78-0348 (BiOBr), the synthesized BN-0.3 exhibits both the AFI framework characteristic of SAPO-5 and the distinctive structure of BiOBr [14,15]. Clear and well-defined diffraction peaks are observed at 2θ values of 7.39°, 12.89°, 19.75°, 20.98°, 22.37°, 25.2°, 31.7°, 39.3°, 46.2°, 50.6° and 57.2°. No additional peaks are present in BN-0.3, indicating that the crystal structure of SAPO-5 and the crystallinity of BiOBr are preserved, with no significant changes in peak positions. The average crystal sizes of the coal gangue-based SAPO-5 molecular sieve and BiOBr were calculated using the Debye–Scherrer equation, yielding values of 52.67 nm and 29.33 nm, respectively.

Figure 2b shows the FT-IR spectra of the N-SAPO-5 molecular sieve, BiOBr, and BN-0.3 nanocomposite. The characteristic peak at 521 cm^−1^ in both BiOBr and BN-0.3 nanocomposite is attributed to the Bi-O bond stretching vibration, while the peak at 1637 cm^−1^ corresponds to the Br-O bond stretching vibration [16,17]. Additionally, the BN-0.3 nanocomposite displays the characteristic peaks of the SAPO-5 molecular sieve, with adsorption peak positions similar to those reported in the literature [11,12,13].

Figure 2c,d show the SEM images of N-SAPO-5 molecular sieve, BiOBr, and BN-0.3 nanocomposite (the EDS spectrum of BN-0.3 nanocomposite is provided in Figure S2). The N-SAPO-5 molecular sieve crystals exhibit clear edges and corners, with a regular hexagonal prism structure [18]. BiOBr has a nanosheet morphology with an average size of 115 nm. Figure 2e demonstrates that BiOBr is well adhered to the N-SAPO-5 molecular sieve, and the composite maintains the original morphology of the materials.

**Figure 3:**
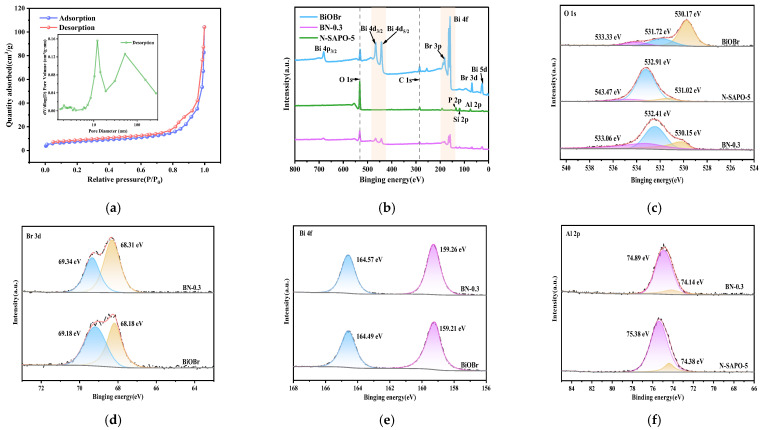
(**a**) N_2_ adsorption–desorption isotherm and pore size distribution of the BN-0.3 nanocomposite; (**b**) XPS survey spectra of N-SAPO-5 molecular sieve, BiOBr, and BN-0.3 nanocomposite; (**c**) High-resolution spectra of O 1s of N-SAPO-5 molecular sieve, BiOBr, and BN-0.3 nanocomposite; (**d**) High-resolution spectra of Br 3d of BiOBr, BN-0.3 nanocomposite; (**e**) High-resolution spectra of Bi 4f of BiOBr, BN-0.3 nanocomposite; (**f**) High-resolution spectra of Al 2p of N-SAPO-5 molecular sieve and BN-0.3 nanocomposite.

Figure 3a shows the N_2_ adsorption–desorption isotherm and pore size distribution of the BN-0.3 nanocomposite. The adsorption–desorption isotherm shows that the BN-0.3 nanocomposite follows a Langmuir type I isotherm and exhibits an H1-type hysteresis loop. Based on the pore size distribution curve and data (Table 1), the average mesopore diameter of the BN-0.3 nanocomposite is 25.19 nm, indicating a uniform mesoporous structure. This pore structure provides a large number of adsorption sites, facilitating the occurrence of monolayer adsorption [11,19].

Figure 3c shows the O 1s high-resolution spectra of N-SAPO-5 molecular sieve, BiOBr, and BN-0.3 nanocomposite. The peaks at 533.06 eV, 532.41 eV, and 530.15 eV correspond to oxygen species adsorbed on oxygen vacancies, hydroxyl groups, and lattice oxygen, respectively [20]. The peaks at 69.34 eV and 68.31 eV correspond to Br 3d_7/2_ and Br 3d_5/2_ (Figure 3d) [21]. In the Bi 4f high-resolution spectrum (Figure 3e), the peaks at 164.57 eV and 159.26 eV correspond to Bi 4f_5/2_ and Bi 4f_7/2_. An increase in the binding energies of Br 3d and Bi 4f was observed in the BN-0.3 nanocomposite, indicating a lower electron cloud density in the BN-0.3 nanocomposite compared to pure BiOBr. The binding energy of Al 2p in the BN-0.3 nanocomposite is reduced (Figure 3f), indicating an increase in the electron cloud density near the Al sites on the surface. This suggests that electrons are transferred from BiOBr to the Al sites of the N-SAPO-5 molecular sieve [12], which facilitates the separation of photogenerated electron-hole pairs in BiOBr and provides a migration path for charge carriers at the heterojunction interface of the BN-0.3 nanocomposite.

### 3.2. Adsorption of MB

#### 3.2.1. Adsorption Kinetics

**Figure 4:**
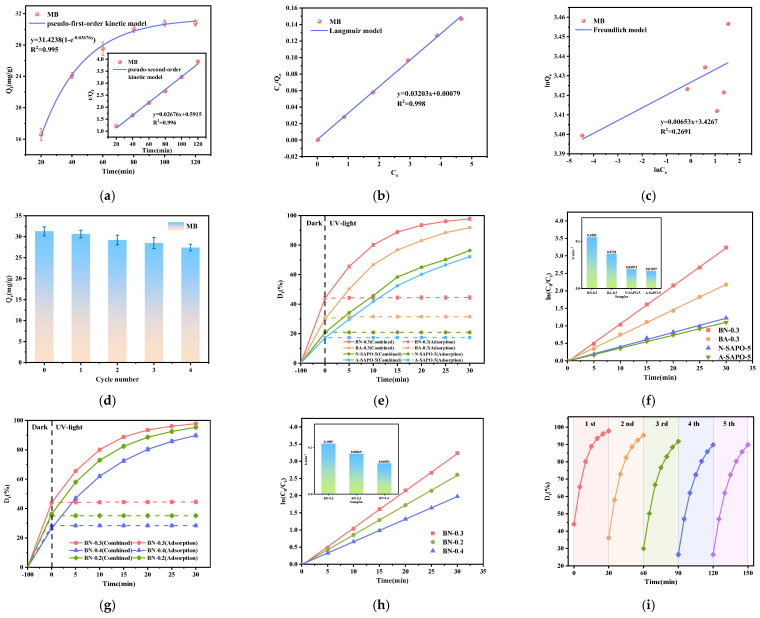
(**a**) Kinetic fitting of MB adsorption; (**b**) Langmuir isothermal fitting of MB adsorption; (**c**) Freundlich isothermal fitting of MB adsorption; (**d**) regeneration performance of the BN-0.3 nanocomposite adsorbent; (**e**) D_t_ ~ t result of MB solution on BN-0.3 nanocomposite, BA-0.3 nanocomposite, N-SAPO-5 molecular sieve and A-SAPO-5 molecular sieve under 300 W Xe lamp; (**f**) ln (C_0_/C_t_) ~ t and first-order kinetics results of MB solution on BN-0.3 nanocomposite, BA-0.3 nanocomposite, N-SAPO-5 molecular sieve and A-SAPO-5 molecular sieve under 300 W Xe lamp; (**g**) D_t_ ~ t result of MB solution on BN-(0.2~0.4) nanocomposite under 300 W Xe lamp; (**h**) ln (C_0_/C_t_) ~ t and first-order kinetics results of MB solution on BN-(0.2~0.4) nanocomposite under 300 W Xe lamp; (**i**) photocatalytic regeneration performance of the BN-0.3 nanocomposite.

The results of the adsorption kinetics fitting are shown in Figure 4a, and Table 2 presents key parameters for both kinetic models. In general, the pseudo-first-order kinetic model is suitable for describing the initial adsorption stage and, thus, exhibits a lower correlation coefficient (R^2^). In the pseudo-second-order kinetic model, the R^2^ value for MB is 0.996, which is closer to 1, indicating that the pseudo-second-order model more accurately describes the adsorption behavior of the BN-0.3 nanocomposite. This suggests that chemical adsorption is the predominant mechanism in the adsorption process, involving electron sharing or transfer between MB and the BN-0.3 nanocomposite [22].

#### 3.2.2. Adsorption Isotherm

Figure 4b,c show the Langmuir and Freundlich isotherm fitting curves for MB adsorption, respectively. Table 3 lists key parameters for both isotherm models. The Freundlich and Langmuir isotherm models are primarily used for analyzing adsorption equilibrium. The Langmuir model for MB adsorption, with an R^2^ value of 0.998, exceeds the R^2^ of the Freundlich model (0.2691), indicating that the adsorption follows a uniform monolayer mechanism [23]. According to the Langmuir model, the maximum calculated Q_e_ value for MB adsorption by the BN-0.3 nanocomposite is 31.22 mg·g^−1^.

#### 3.2.3. Regeneration Performance of the Adsorbent

Figure 4d illustrates the adsorption cycling performance of the BN-0.3 nanocomposite. With increasing cycles, the MB adsorption capacity slightly decreases. After five cycles, the MB adsorption capacity remains as high as 27.56 mg·g^−1^, indicating that the prepared BN-0.3 nanocomposite exhibits excellent cyclic adsorption performance.

### 3.3. Combined Adsorption and Photocatalytic Removal

The photocatalytic reaction was conducted in a photochemical reactor, with cooling water circulated to maintain a constant temperature. Adsorption was performed under ambient conditions, and the removal efficiency of MB was used to evaluate the combined adsorption and photocatalytic removal performance of the samples.

Figure 4e examines the effect of loading on the MB adsorption-photocatalytic degradation performance. All samples reached adsorption–desorption equilibrium within 100 min, and no significant change in the adsorption removal rate was observed after 100 min. All samples exhibited photocatalytic degradation performance upon exposure to light. The BN-0.3 nanocomposite achieved MB removal rate of 97.8% and MB mineralization rate of 57.4% (Figure S3) within 30 min. The degradation process of the sample follows first-order kinetics, with the first-order kinetic fitting and rate constant shown in Figure 4f. BN-0.3 nanocomposite (0.1085 min^−1^) exhibits the highest photocatalytic activity, being 1.5 times that of BA-0.3 nanocomposite (0.0728 min^−1^), 1.68 times that of BiOBr (0.06453 min^−1^) (Figure S4), 2.66 times that of N-SAPO-5 molecular sieve (0.04074 min^−1^), and 2.94 times that of A-SAPO-5 molecular sieve (0.03699 min^−1^). Therefore, the incorporation of BiOBr and the use of N-SAPO-5 molecular sieve as the support significantly enhance the photocatalytic performance of the nanocomposite.

Therefore, under the condition of using the N-SAPO-5 molecular sieve as the support, BN-(0.2~0.4) nanocomposite composites were prepared by varying the BiOBr loading. The adsorption-photocatalytic performance and first-order kinetic fitting were investigated, as shown in Figure 4g,h. Similarly, all samples reached adsorption–desorption equilibrium within 100 min, with no significant change in the adsorption removal rate after this period. Upon light exposure, all BN-(0.2~0.4) nanocomposites demonstrated good photocatalytic performance. The MB degradation rates within 30 min were 95.3%, 97.8%, and 89.8%, respectively. Among them, the BN-0.3 (0.1085 min^−1^) nanocomposite exhibited the best combined adsorption-photocatalytic performance. As the BiOBr loading increases, the degradation rate initially rises and then decreases. This trend can be attributed to the following reasons: within a certain range, an increase in BiOBr loading enhances the number of active sites, leading to the generation of more reactive species that accelerate the photocatalytic reaction. However, when the loading exceeds a certain threshold, aggregation occurs within the solution, hindering effective contact between reactants and active sites. Moreover, aggregation causes light scattering and shielding, which reduces light utilization and slows down the reaction [15].

Figure 4i shows the photocatalytic cycling performance of the BN-0.3 nanocomposite. With increasing cycles, the MB degradation rate gradually decreases. After five cycles, the MB degradation rate remained at 92%, demonstrating that the BN-0.3 nanocomposite possesses excellent reusability.

**Figure 5:**
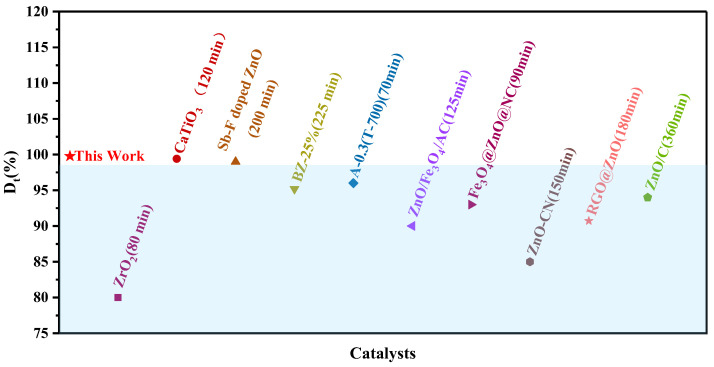
Comparison of MB removal efficiencies of catalysts under light irradiation.

The photocatalytic performance of the BN-0.3 nanocomposite for MB degradation is compared with that of other catalysts in Figure 5 [7,8,24,25,26,27,28,29,30,31]. The removal rate within 30 min is significantly higher than that of other catalysts. The results indicate that the BN-0.3 nanocomposite exhibits strong photocatalytic performance, demonstrating promising potential for methylene blue degradation in real wastewater applications.

### 3.4. Optical and Electrochemical Characterization of BiOBr/Coal Gangue-Based SAPO-5 Nanocomposite

Since BN-0.3 nanocomposite exhibits the best photocatalytic performance, BN-0.3 nanocomposite, BiOBr, and N-SAPO-5 molecular sieve were selected for optical and electrochemical characterization.

**Figure 6:**
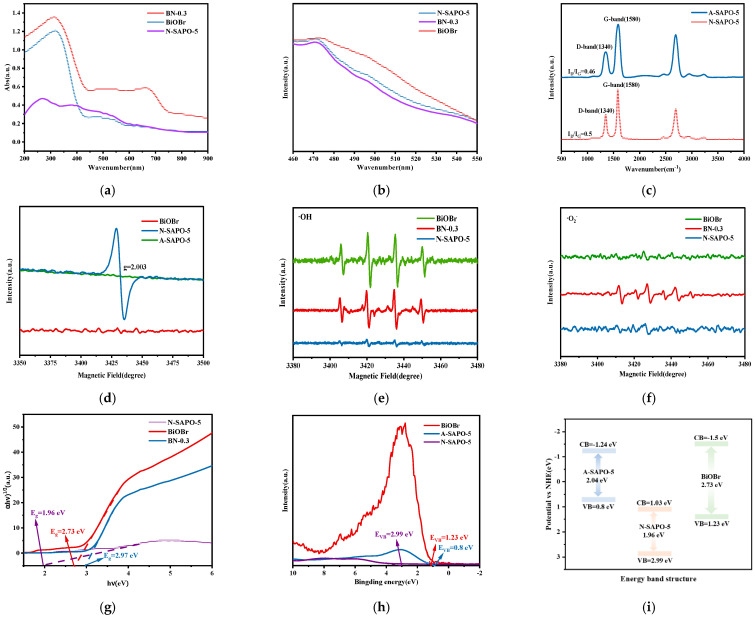
(**a**) UV–visible DRS spectra of BN-0.3 nanocomposite, BiOBr, N-SAPO-5 molecular sieve; (**b**) PL emission spectra of BN-0.3 nanocomposite, BiOBr, N-SAPO-5 molecular sieve; (**c**) Raman spectra of N-SAPO-5 molecular sieve, A-SAPO-5 molecular sieve; (**d**) EPR spectra of oxygen vacancies in N-SAPO-5 molecular sieve and A-SAPO-5 molecular sieves. (**e**) EPR of DMPO-•OH of BN-0.3 nanocomposite, N-SAPO-5 molecular sieve; (**f**) EPR of DMPO-•O_2_^−^ of BN-0.3 nanocomposite, N-SAPO-5 molecular sieve; (**g**) Tauc plots of (αhν)^1/2^ of BN-0.3 nanocomposite, BiOBr, N-SAPO-5 molecular sieve (**h**) VB-XPS Spectral Plot of BN-0.3 nanocomposite, A-SAPO-5 molecular sieve, N-SAPO-5 molecular sieve; (**i**) band structure diagram of the BN-0.3 nanocomposite, A-SAPO-5 molecular sieve, N-SAPO-5 molecular sieve.

Figure 6a shows the UV-Vis diffuse reflectance spectra of BN-0.3 nanocomposite, BiOBr, and N-SAPO-5 molecular sieve. Within the range of 200~800 nm, BiOBr exhibits an absorption edge at 440 nm, while the N-SAPO-5 molecular sieve demonstrates light absorption across the entire spectrum, although with relatively low intensity. Upon the formation of the BN-0.3 nanocomposite, the absorbance significantly increased, indicating that BN-0.3 nanocomposite exhibits enhanced light absorption capability. Furthermore, its absorption edge is broader compared to BiOBr, which is beneficial for the generation of photoinduced charge carriers [32].

Figure 6b presents the photoluminescence (PL) emission spectra of BN-0.3 nanocomposite, BiOBr, and N-SAPO-5 molecular sieve, which are used to evaluate the efficiency of photogenerated charge carrier separation in the materials. The BN-0.3 nanocomposite exhibits a lower PL intensity, indicating a reduced recombination rate of photogenerated charge carriers, allowing more electrons and holes to participate in redox reactions [33].

Considering that the BA-0.3 nanocomposite also demonstrates favorable photocatalytic performance, it was studied in conjunction with the BN-0.3 nanocomposite. Figure 6c presents the Raman spectra of the N-SAPO-5 molecular sieve and the A-SAPO-5 molecular sieve molecular sieves. The D-band, appearing at 1340 cm^−1^, is associated with the disordered structure in carbon materials and signifies defects within the carbon structure. The G-band, located at 1580 cm^−1^, arises from the in-plane vibrations of sp^2^-hybridized carbon atoms and reflects the graphitization degree of the carbon material [8]. The disorder degree of the material is evaluated by the intensity ratio of the D-band to the G-band (I_D_/I_G_). The I_D_/I_G_ ratio of the N-SAPO-5 molecular sieve (0.5) is higher than that of the A-SAPO-5 molecular sieve (0.46), indicating that the N-SAPO-5 molecular sieve exhibits a greater number of structural defects compared to the A-SAPO-5 molecular sieve.

Based on the photocatalytic performance evaluation and Raman spectroscopy analysis, it can be inferred that the N-SAPO-5 molecular sieve contains more structural defects. Therefore, EPR characterization of the N-SAPO-5 molecular sieve, the A-SAPO-5 molecular sieves, and BiOBr were conducted, as shown in Figure 6d. The N-SAPO-5 molecular sieve exhibits a peak at a g-value of 2.003 [34], indicating the presence of oxygen vacancies. In contrast, the oxygen vacancy signal is less pronounced in the A-SAPO-5 molecular sieve and BiOBr, suggesting that the N_2_ calcination process promotes the incorporation of oxygen vacancies, indicating that the N_2_ calcination process promotes the incorporation of oxygen vacancies, which suggests that the oxygen vacancies in the BN-0.3 nanocomposite are provided by the N-SAPO-5 molecular sieve. The increase in oxygen vacancies contributes to the enhancement of photocatalytic performance.

EPR spectroscopy was further used to characterize the types and intensities of radicals generated during the photocatalytic process, as shown in Figure 6e,f. Under illumination, two distinct peak patterns, 1:2:2:1 and 1:1:1:1, were clearly observed. The intensity of hydroxyl radicals generated by BiOBr under illumination is the highest, while the intensity of hydroxyl radicals generated by the BN-0.3 nanocomposite is significantly higher than that of the N-SAPO-5 molecular sieve. For superoxide radicals, the intensity generated by the BN-0.3 nanocomposite is higher than that of BiOBr and the N-SAPO-5 molecular sieve. This indicates that the BN-0.3 nanocomposite generates ·OH and ·O_2_⁻ radicals under illumination [35,36], with significantly high generation intensities. To further identify the active species that have the greatest impact on the photocatalytic process, t-BuOH (·OH), TEOA (h^+^), and BQ (·O_2_^−^) were used as scavengers to eliminate reactive species, as shown in Figure S5. Among these, t-BuOH exhibited the most significant inhibitory effect, followed by TEOA. This suggests that ·OH plays a dominant role in the photocatalytic process.

### 3.5. Band Structure and Photocatalytic Mechanism

The band gap was calculated according to Equation (3) [37], and the Tauc plot is shown in Figure 6g.

*(αhν)^n^* = *A (hν − E_g_)* (3)

In the equation, *α* represents the absorption coefficient due to diffusion, h is Planck’s constant, *ν* is the photon frequency, and *n* = 1/2.

The band gap energies (E_g_) of BiOBr, BN-0.3 nanocomposite, N-SAPO-5 molecular sieve, and A-SAPO-5 molecular sieve were determined through calculations, as shown in Figure 6g and Figure S6. The values are 2.73 eV, 2.97 eV, 1.96 eV, and 2.04 eV, respectively. Based on the VB-XPS results (Figure 6h), the valence band potentials (E_VB_) of BiOBr, N-SAPO-5 molecular sieve, and A-SAPO-5 molecular sieve are 1.23 eV, 2.99 eV, and 0.8 eV, respectively.

According to the empirical relation given by Equation (4):

*E_CB_* = *E_VB_* − *E_g_* (4)

In the equation, *E_CB_*, *E_VB_* and *E_g_* represent the conduction band, valence band, and band gap, respectively. The conduction band potentials (*E_CB_*) of BiOBr, N-SAPO-5 molecular sieve, and A-SAPO-5 molecular sieve are −1.5 eV, 1.03 eV, and −1.24 eV, respectively. Based on the positions of the valence and conduction bands, a Z-Type heterojunction is formed between BiOBr and N-SAPO-5 molecular sieve. The band structure diagram is shown in Figure 6i.

BiOBr/N-SAPO-5 + hν→BiOBr/N-SAPO-5(h^+^ + e^−^) (5)

BiOBr/N-SAPO-5(h^+^ + e^−^)→BiOBr(e^−^)/N-SAPO-5(h^+^) (6)

BiOBr/N-SAPO-5 + MB + e^−^→BiOBr/N-SAPO-5-MB_adsorption_^−*^ (7)

BiOBr(e^−^)/N-SAPO-5(h^+^) + H_2_O/O_2_/-OH→OH/O_2_^−^ (8)

MB_adsorption_^−*^+·OH/h^+^→Degradation products (9)

**Figure 7:**
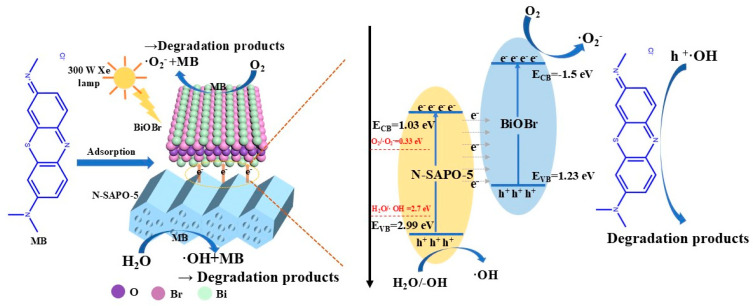
Schematic of the photocatalytic reaction mechanism.

This study presents the mechanism of the photocatalytic process, illustrated in Figure 7. The photocatalytic process can be described by Equations (5)–(9). During the adsorption process, π-π interactions occur between the aromatic structure of the dye molecules and the graphite structure on the surface of the carbon material, leading to chemisorption, which activates MB and facilitates the subsequent photocatalytic reaction [8]. The illumination is conducive to the generation of electron-hole pairs in both N-SAPO-5 molecular sieve and BiOBr. The heterojunction formed between the N-SAPO-5 molecular sieve and BiOBr promotes the separation and transfer of photogenerated electrons and holes. If the energy band alignment between N-SAPO-5 molecular sieve and BiOBr forms a Type II heterojunction, according to the principle of energy level differences, electrons from the conduction band (CB) of BiOBr transfer to the CB of N-SAPO-5 molecular sieve, while holes from the valence band (VB) of N-SAPO-5 molecular sieve move to the VB of BiOBr. However, the VB potential of BiOBr is lower than that of H_2_O/·OH (2.7 eV), indicating that the holes in the VB of BiOBr cannot oxidize H_2_O to produce ·OH. Therefore, the Type II heterojunction scheme is not correct, and based on this, a Z-type heterojunction scheme is proposed.

When the BiOBr/N-SAPO-5 molecular sieve composite system is illuminated, both the N-SAPO-5 molecular sieve and BiOBr absorb photons, causing electrons (e^−^) to transition from the VB to the CB, leaving behind h^+^. Subsequently, due to the strong electrostatic attraction between the e^−^ in the CB of N-SAPO-5 molecular sieve and the h^+^ in the VB of BiOBr, and the smaller energy level difference between them compared to the internal energy level difference within BiOBr itself, electrons in the CB of N-SAPO-5 are more likely to recombine with holes in the VB of BiOBr. As a result, most of the e^−^ remain in the CB of BiOBr, while most of the h^+^ remain in the N-SAPO-5 molecular sieve, leading to the effective separation of e^−^ and h^+^. The MB adsorbed on the catalyst surface (MB _adsorption_) is activated by the electrons on BiOBr, forming activated MB (MB _adsorption_ ^−*^). Oxygen (O_2_) captures electrons from the CB of BiOBr, generating ·O_2_^−^, while -OH/H_2_O undergoes oxidation to form ·OH under the influence of the holes enriched in the VB of N-SAPO-5 molecular sieve. Finally, ·OH and ·O_2_^−^ jointly participate in the degradation of MB _adsorption_ ^−*^. Based on the results of quenching experiments, it can be concluded that h^+^ and ·OH play a dominant role in the photocatalytic degradation of MB.

## 4. Conclusions

This study used coal gangue as the raw material to prepare an oxygen vacancy-rich coal gangue-based SAPO-5 molecular sieve using hydrothermal synthesis and N_2_ calcination. The BiOBr/coal gangue-based SAPO-5 nanocomposite was then synthesized by incorporating BiOBr. When the BiOBr loading is 30%, the highest photocatalytic degradation rate can reach 97.8%, and the mineralization rate can reach 57.4%. The adsorption process follows the pseudo-second-order kinetic model and Langmuir adsorption isotherm, while the photocatalytic process adheres to the first-order kinetic model. The photocatalytic mechanism of the BiOBr/coal gangue-based SAPO-5 nanocomposite is proposed. The Z-type heterojunction formed between BiOBr and SAPO-5 molecular sieve facilitates the effective separation of photogenerated charge carriers, thereby enhancing the redox capacity. This study expands the potential high-value applications of coal gangue and offers a simple, efficient, and environmentally friendly method for the removal of organic pollutants.

## Figures and Tables

**Table 1:** Pore size distribution of CG and BN-0.3 nanocomposite.

Sample	S_BET_/(m^2^·g^−1^)	Total Pore Volume/(cm^3^·g^−1^)	Average Mesoporous Pore Size/(nm)
CG	2.04	--	--
BN-0.3 nanocomposite	25.83	0.099	25.19

**Table 2:** Kinetic model parameters for adsorption of MB onto BN-0.3 nanocomposite.

	Pseudo-First-Order Model			Pseudo-Second-Order Model		
	K_1_/min^−1^	Q_e_/(mg·g^−1^)	R^2^	K_2_/(g·mg^−1^·min^−1^)	Q_e_/(mg·g^−1^)	R^2^
MB	0.03676	31.4238	0.995	0.00121	37.37	0.996

**Table 3:** Isothermal parameters of Langmuir and Freundlich models for the adsorption of MB onto BN-0.3 nanocomposite.

	Langmuir				Freundlich		
	K_L_/(L·mg^−1^)	Q_m_/(mg·g^−1^)	R^2^		K_F_/((mg·g^−1^)·(L·mg^−1^)1/n)	1/n	R^2^
MB	40.55	31.22	0.998		25.73	0.00653	0.2691

## Data Availability

The original contributions presented in this study are included in the article/Supplementary Material.

## Supplementary Materials

The following supporting information can be downloaded at: https://www.mdpi.com/article/10.3390/nano15050321/s1. Table S1. Chemical compositions of CG and PCG. Figure S1. (a) The effect of adsorbent dosage on the adsorption performance of MB; (b) The effect of initial solution concentration on the adsorption performance of MB; (c) The effect of pH on MB adsorption capacity; (d) The effect of photocatalyst dosage on MB removal rate; (e) The effect of initial solution concentration on MB removal rate; (f) The effect of pH value on the photocatalytic removal rate of MB. Figure S2. EDS spectrum of BN-0.3 nanocomposite. Figure S3. Total carbon (TC) and total organic carbon (TOC) content in MB solution before and after photocatalytic reaction. Figure S4. (a) D_t_ ~ t result of MB solution on BiOBr under 300 W Xe lamp; (b) ln (C_0_/C_t_) ~ t and first-order kinetics results of MB solution on BiOBr under 300 W Xe lamp. Figure S5. D_t_ ~ t curves of MB solution on BN-0.3 nanocomposite with the presence of different scavengers. Figure S6. Tauc plots of (αhν)^1/2^ of A-SAPO-5 molecular sieve.
