# Supplementary material for: BiOBr/Coal Gangue-Based SAPO-5 Molecular Sieve Nanocomposite for Enhanced Adsorption and Photocatalytic Degradation of Methylene Blue
Source: Nanomaterials (Basel). 2025 Feb 20;15(5):321. doi: 10.3390/nano15050321 (PMC11902256; doi:10.3390/nano15050321)
Supplement: Supplementary file 1 [file nanomaterials-15-00321-s001.zip › nanomaterials-3407840-supplementary.pdf]

BiOBr/Coal gangue-based SAPO-5 molecular sieve nanocomposite for enhanced adsorption and photocatalytic degradation of methylene blue

Boyang Xu[1], Jie Chen[1,*], Kai Wang[2], Pengfei Li[1], Le Kang[1,*], Huiling Du[1], Qianqian Liu[1], Xiaoqing Lian[1]

1 College of Materials Science and Engineering, Xi'an University of Science and Technology, Xi'an, 710054, China

2 School of Electrical Engineering, Qingdao University, Qingdao 266071, China

Table S1. Chemical compositions of CG and PCG.

Compositions	SiO_2	Al_2O_3	MgO	Na_2O	K_2O	CaO	TiO_2	Fe_2O_3	LOI
CG (wt%)	64.58	22.01	0.06	1.01	3.29	1.47	5.58	1.15	0.75
PCG (wt%)	64.50	18.96	1.65	1.70	4.21	0.92	1.15	5.89	0.46

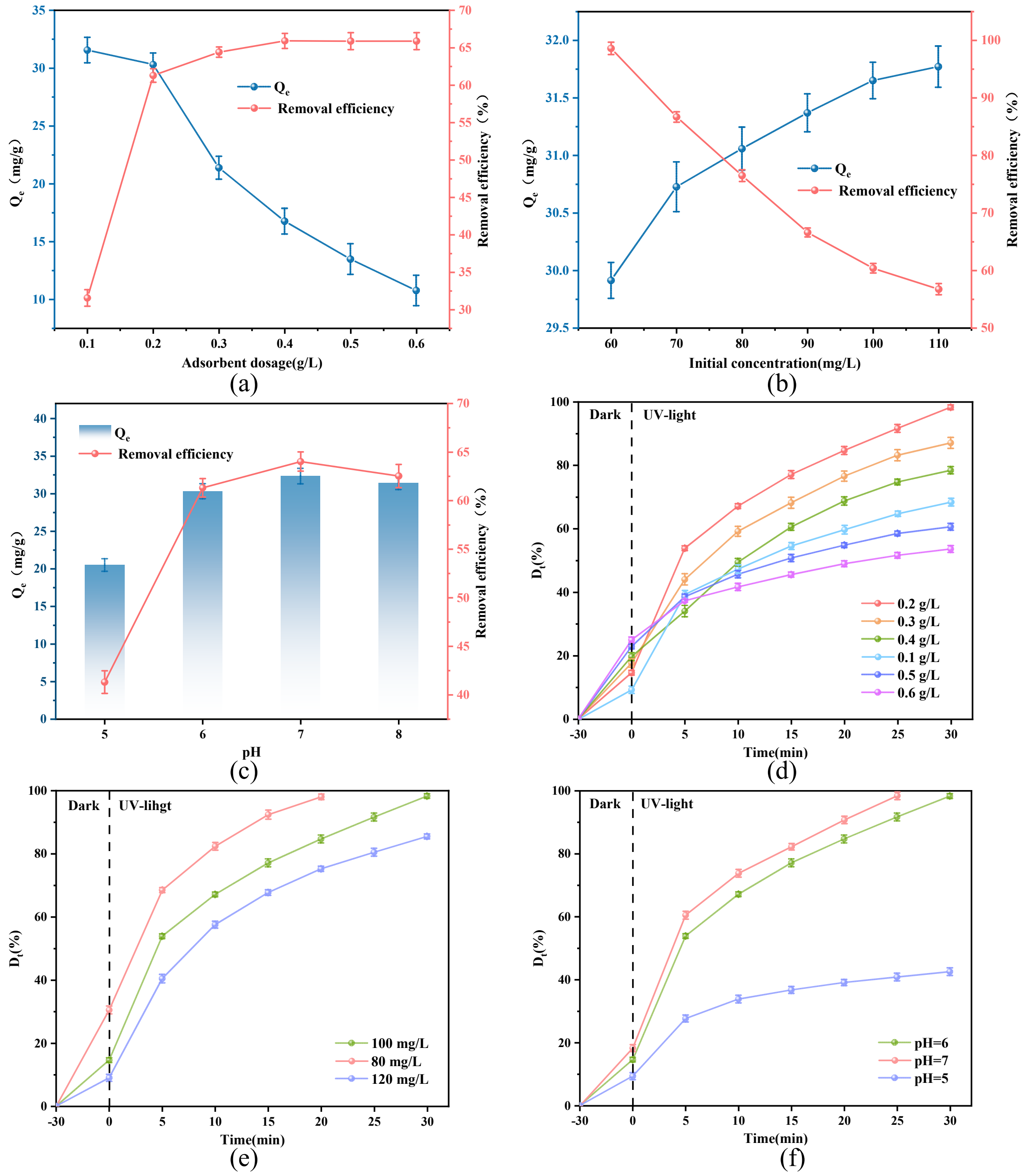

Figure S1 (a) The effect of adsorbent dosage on the adsorption performance of MB; (b) The effect of initial solution concentration on the adsorption performance of MB; (c) The effect of pH on MB adsorption capacity; (d) The effect of photocatalyst dosage on MB removal rate; (e) The effect of initial solution concentration on MB removal rate; (f) The effect of pH value on the photocatalytic removal rate of MB

The general expression Q_e ($mg \cdot g^{-1}$) for calculating adsorption capacity is:

$$Q_e = \frac{(C_0 - C_e)V}{m} \tag{S1}$$

The adsorption capacity Q_t ($mg \cdot g^{-1}$) at different times is:

$$Q_t = \frac{(C_0 - C_t)V}{m} \tag{S2}$$

In equation (S1-S2), Q_e ($mg \cdot g^{-1}$) and Q_t ($mg \cdot g^{-1}$) respectively represent the equilibrium adsorption capacity of MB and the adsorption capacity at time t; C_0($mg \cdot L^{-1}$), C_t($mg \cdot L^{-1}$) and Ce(mg·L-1) represent the initial concentration of the adsorption solution, the concentration at time t, and the concentration at adsorption equilibrium, respectively; V (L) represents the volume of the adsorption solution; m (g) is the amount of molecular sieve used.

Lagergren pseudo first order kinetic model is generally expressed as:

$$\ln\left(Q_e - Q_t\right) = \ln Q_e - K_1 t \tag{S3}$$

Taking the logarithm on both sides yields:

$$Q_t = Q_e(1 - e^{-K_1 t}) \tag{S4}$$

Lagergren pseudo second order kinetic model is generally expressed as:

$$\frac{t}{Q_t} = \frac{t}{Q_e} + \frac{1}{K_2 Qe^2} \tag{S5}$$

In equation (S5-S6), K_1 (min^{-1}) and K_2 ($g/(mg \cdot min)$) represent the adsorption rate constants of the pseudo first order kinetic model and the pseudo second order kinetic model, respectively.

The Langmuir model for adsorption isotherms is generally expressed as:

$$\frac{C_e}{Q_e} = \frac{C_e}{Q_m} + \frac{1}{Q_m K_L} \tag{S6}$$

The Freundlich model for adsorption isotherms is generally expressed as:

$$\ln Q_e = \left(\frac{1}{n}\right) \ln C_e + \ln K_F \tag{S7}$$

In equation (S6-S7), Q_m ($mg \cdot g^{-1}$) is the maximum adsorption capacity, and K_L ($L \cdot mg^{-1}$) is the Langmuir model adsorption equilibrium constant, K_F is the fitting coefficient of the Freundlich model, 1/n is the adsorption index of the Freundlich model. When $0.10<1/n<0.50$, it is easy to adsorb, Difficult to adsorb when $1/n>2$.

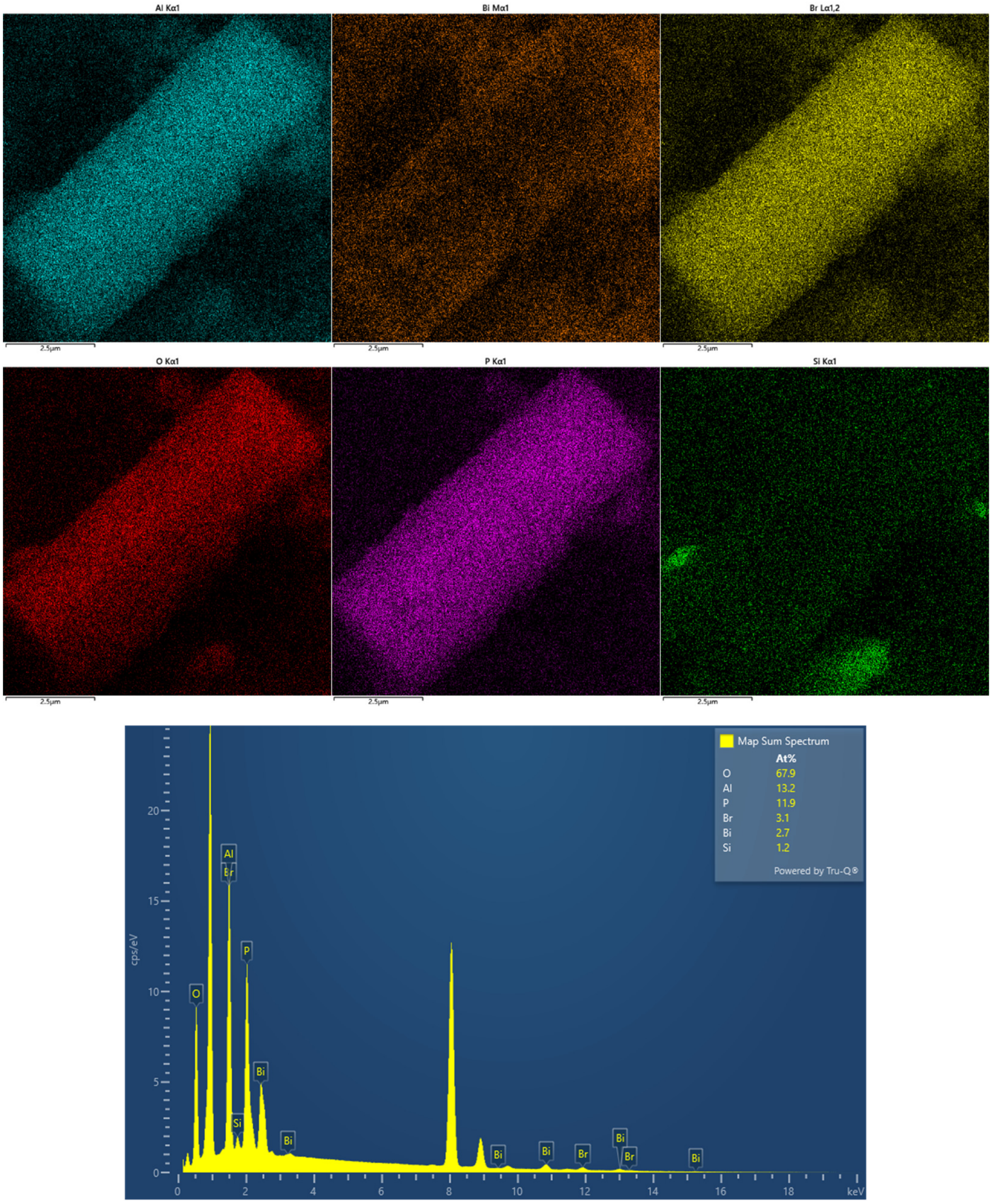

Figure S2 EDS spectrum of BN-0.3 nanocomposite

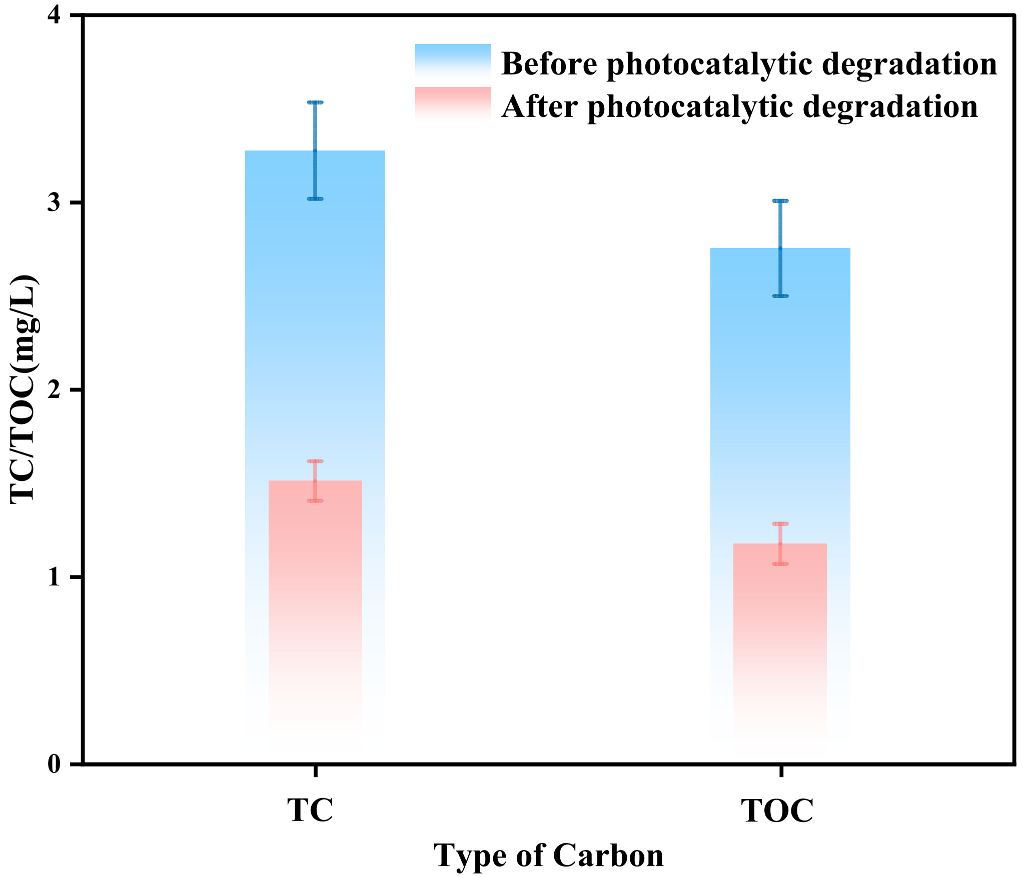

Figure S3 Total carbon (TC) and total organic carbon (TOC) content in MB solution before and after photocatalytic reaction

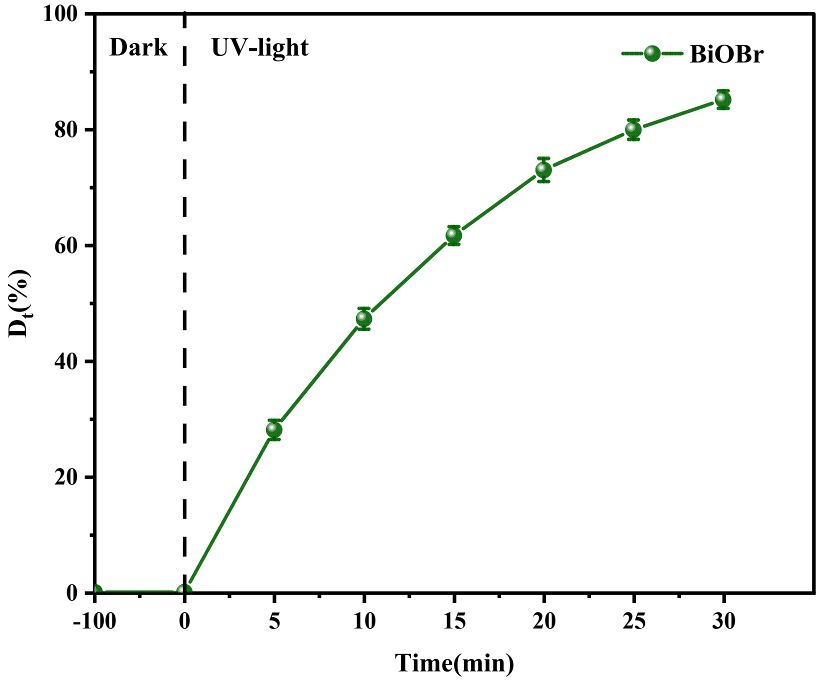

(a)

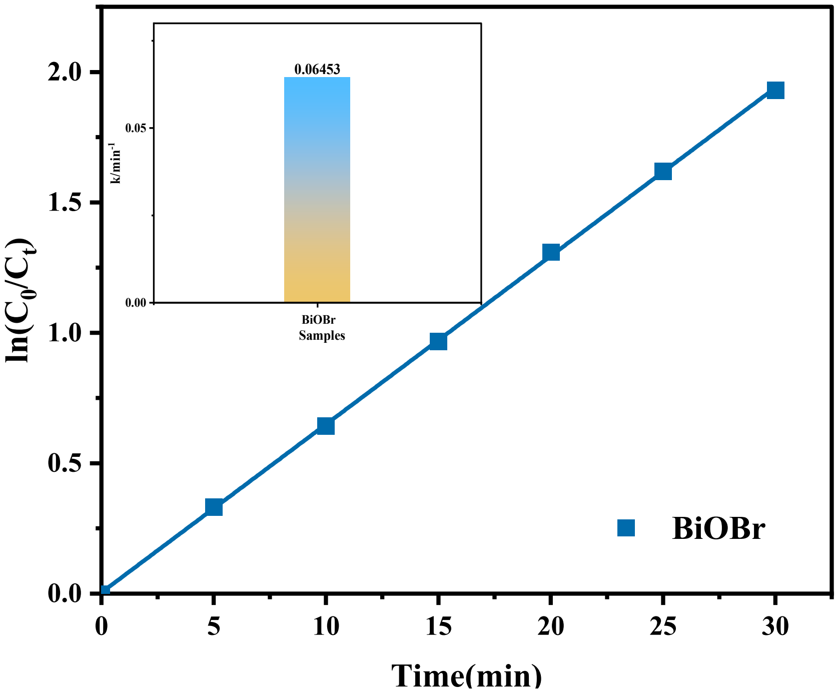

(b)

Figure S4 (a) D_t ~ t result of MB solution on BiOBr under 300 W Xe lamp; (b) ln (C_0/C_t) ~ t and first-order kinetics results of MB solution on BiOBr under 300 W Xe lamp;

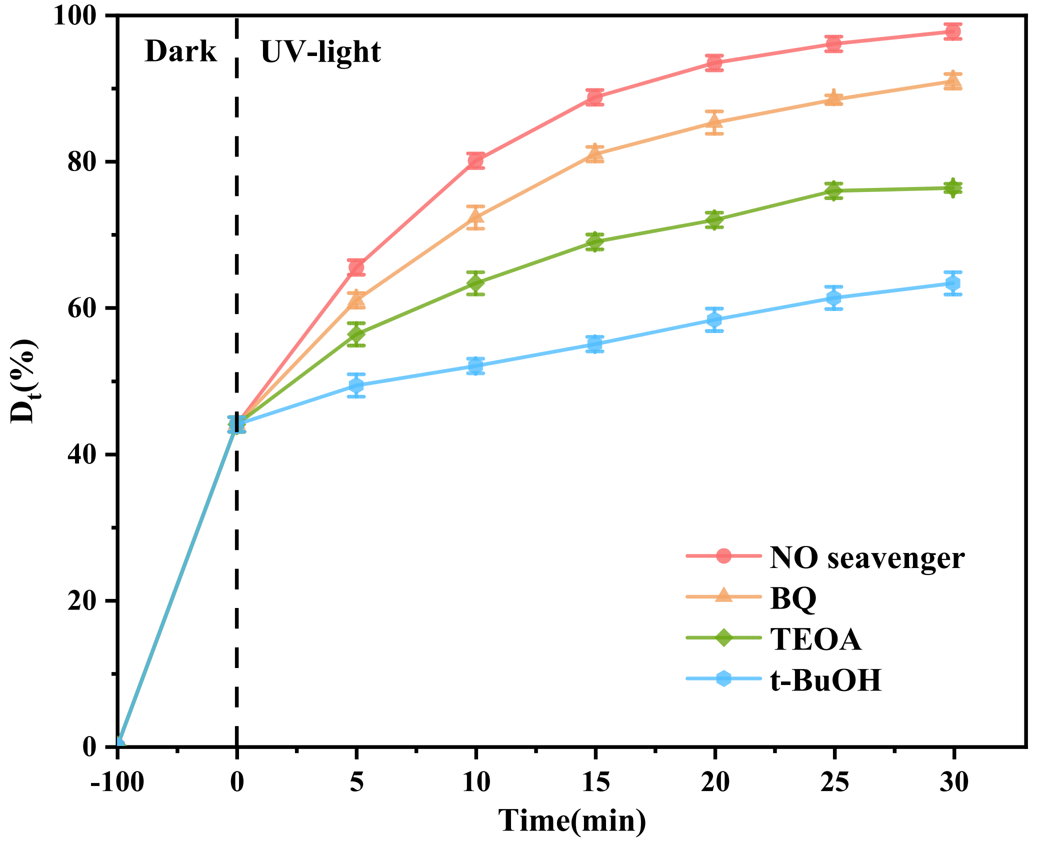

Figure S5 $D_t \sim t$ curves of MB solution on BN-0.3 nanocomposite with the presence of different scavengers

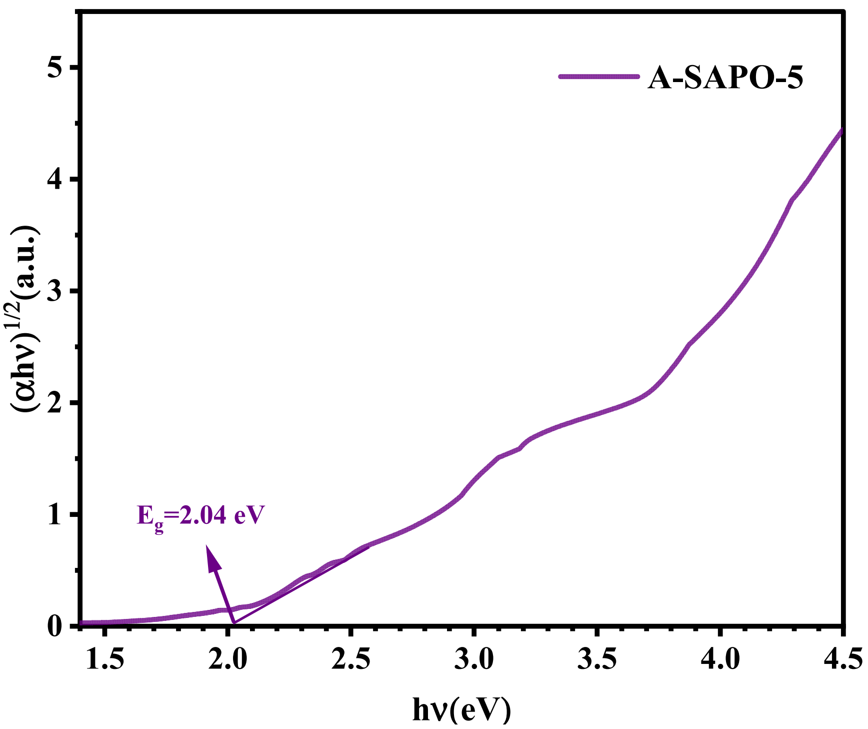

Figure S6 Tauc plots of $(\alpha h\nu)^{1/2}$ of A-SAPO-5 molecular sieve
